# MCT1 and MCT4 Expression and Lactate Flux Activity Increase During White and Brown Adipogenesis and Impact Adipocyte Metabolism

**DOI:** 10.1038/s41598-017-13298-z

**Published:** 2017-10-12

**Authors:** Charlotte Petersen, Mette D. Nielsen, Elise S. Andersen, Astrid L. Basse, Marie S. Isidor, Lasse K. Markussen, Birgitte M. Viuff, Ian H. Lambert, Jacob B. Hansen, Stine F. Pedersen

**Affiliations:** 1 Section for Cell Biology and Physiology, Department of Biology, Faculty of Science, University of Copenhagen, Copenhagen, Denmark; 2 Section for Molecular Disease Biology, Department of Veterinary Disease Biology, Faculty of Health and Medical Sciences, University of Copenhagen, Copenhagen, Denmark

## Abstract

Adipose tissue takes up glucose and releases lactate, thereby contributing significantly to systemic glucose and lactate homeostasis. This implies the necessity of upregulation of net acid and lactate flux capacity during adipocyte differentiation and function. However, the regulation of lactate- and acid/base transporters in adipocytes is poorly understood. Here, we tested the hypothesis that adipocyte thermogenesis, browning and differentiation are associated with an upregulation of plasma membrane lactate and acid/base transport capacity that in turn is important for adipocyte metabolism. The mRNA and protein levels of the lactate-H^+^ transporter MCT1 and the Na^+^,HCO_3_^−^ cotransporter NBCe1 were upregulated in mouse interscapular brown and inguinal white adipose tissue upon cold induction of thermogenesis and browning. MCT1, MCT4, and NBCe1 were furthermore strongly upregulated at the mRNA and protein level upon differentiation of cultured pre-adipocytes. Adipocyte differentiation was accompanied by increased plasma membrane lactate flux capacity, which was reduced by MCT inhibition and by MCT1 knockdown. Finally, in differentiated brown adipocytes, glycolysis (assessed as ECAR), and after noradrenergic stimulation also oxidative metabolism (OCR), was decreased by MCT inhibition. We suggest that upregulation of MCT1- and MCT4-mediated lactate flux capacity and NBCe1-mediated HCO_3_^−^/pH homeostasis are important for the physiological function of mature adipocytes.

## Introduction

The continuous increase in the world-wide incidence of obesity and associated diseases including diabetes, cardiovascular disease and cancer have, in conjunction with the known contributions of fat-accumulation-induced adipose dysfunctions to metabolic diseases, spurred renewed interest in the cell biology of the adipose organ^1–3^. Adipose tissue plays pivotal roles in the control of body energy balance, glucose homeostasis and insulin signaling^3–8^. Its defining components are white and brown adipocytes, constituting, respectively, the white (WAT) and brown (BAT) adipose tissue. Whereas WAT stores fat as triglycerides (TAGs), BAT dissipates energy as heat via uncoupling protein 1 (UCP1)-mediated uncoupling of the mitochondrial H^+^ gradient^9^.

White adipocytes are primarily glycolytic even in the presence of oxygen, and hence produce and release large amounts of lactate, as shown in adipocytes *in vitro* as well as in rats and humans *in vivo* ^10–12^. Released lactate is subsequently oxidized in metabolic tissues, or is utilized in hepatic gluconeogenesis^13^. Glucose uptake by white adipocytes *in vitro* exceeds their need for use in TAG production^11^, and it has been proposed that WAT-mediated conversion of glucose to lactate could serve a protective role in counteracting hyperglycemia, a process potentially important in preserving insulin sensitivity^11,14^. Importantly, changes in WAT-mediated lactate homeostasis have been linked to metabolic disturbances such as obesity and diabetes^6,10–12^. Basal lactate production from WAT is increased in obese humans, due to the increased WAT mass^12^. Moreover, diabetes is associated with WAT mitochondrial dysfunction, which is suggested to be compensated by a glycolytic shift^14^ and hence presumably an increased need for lactate efflux. Much less is known about these processes in brown adipocytes, however, also BAT has been shown to contribute significantly to the regulation of plasma glucose levels^15,16^. Finally, recent studies have proposed that lactate production contributes to browning of WAT via a redox-dependent process involving mitochondrial lactate oxidation^17^.

Thus, lactate transport is central to adipocyte function, yet its regulation and roles in this context are incompletely understood. Lactate transport across the plasma membrane is primarily mediated by lactate-H^+^ cotransporters of the SLC16 monocarboxylate carrier family^18^. In most cells, MCT1 (SLC16A1) and MCT4 (SLC16A3) are the predominant lactate uptake- and efflux pathways^19,20^. MCT1 has high, and MCT4 low L-lactate affinity (Km ~3.5–10 and ~22–28 mmol/L, respectively)^19,20^. MCT1 is therefore particularly well suited for inward lactate transport in oxidative cells and MCT4 for export of lactate from glycolytic cells, although the direction of transport is obviously determined by the net driving force for lactate and H^+^. Existing data on lactate transporter expression in adipocytes are scant and to some extent contradictory^17,21–23^.

Here, we reasoned that if lactate flux is increased during adipocyte differentiation, MCT expression and flux capacity are likely to increase during this process and be important for adipocyte metabolism. Phosphofructokinase-1 (PFK-1) activity, the rate-limiting step in glycolysis, is strongly inhibited at acidic cellular pH^24^. Since cellular pH is continually challenged by the CO_2_ and H^+^ produced during mitochondrial respiration and ATP hydrolysis, respectively^25^, we also asked whether bicarbonate transporters are upregulated during adipocyte differentiation.

We report that MCT1 and the Na^+^,HCO_3_^−^ cotransporter NBCe1 are upregulated upon cold-induced thermogenesis (interscapular BAT, iBAT) and browning (inguinal WAT, iWAT) in mice *in vivo*. Further, MCT1 and MCT4 and NBCe1 are upregulated at the mRNA and protein level during white and brown adipocyte differentiation *in vitro*. Differentiation-induced MCT1 and MCT4 upregulation is accompanied by a marked increase in lactate flux capacity, predominately mediated by MCT1, and MCT inhibition strongly attenuates β-adrenergically induced metabolic activity. Collectively, these data point to important roles for acid-base transport in adipocyte function.

## Results

### Relative mRNA levels of MCT1 and NBCe1 are increased in iBAT and iWAT upon cold exposure *in vivo*

**Figure 1:**
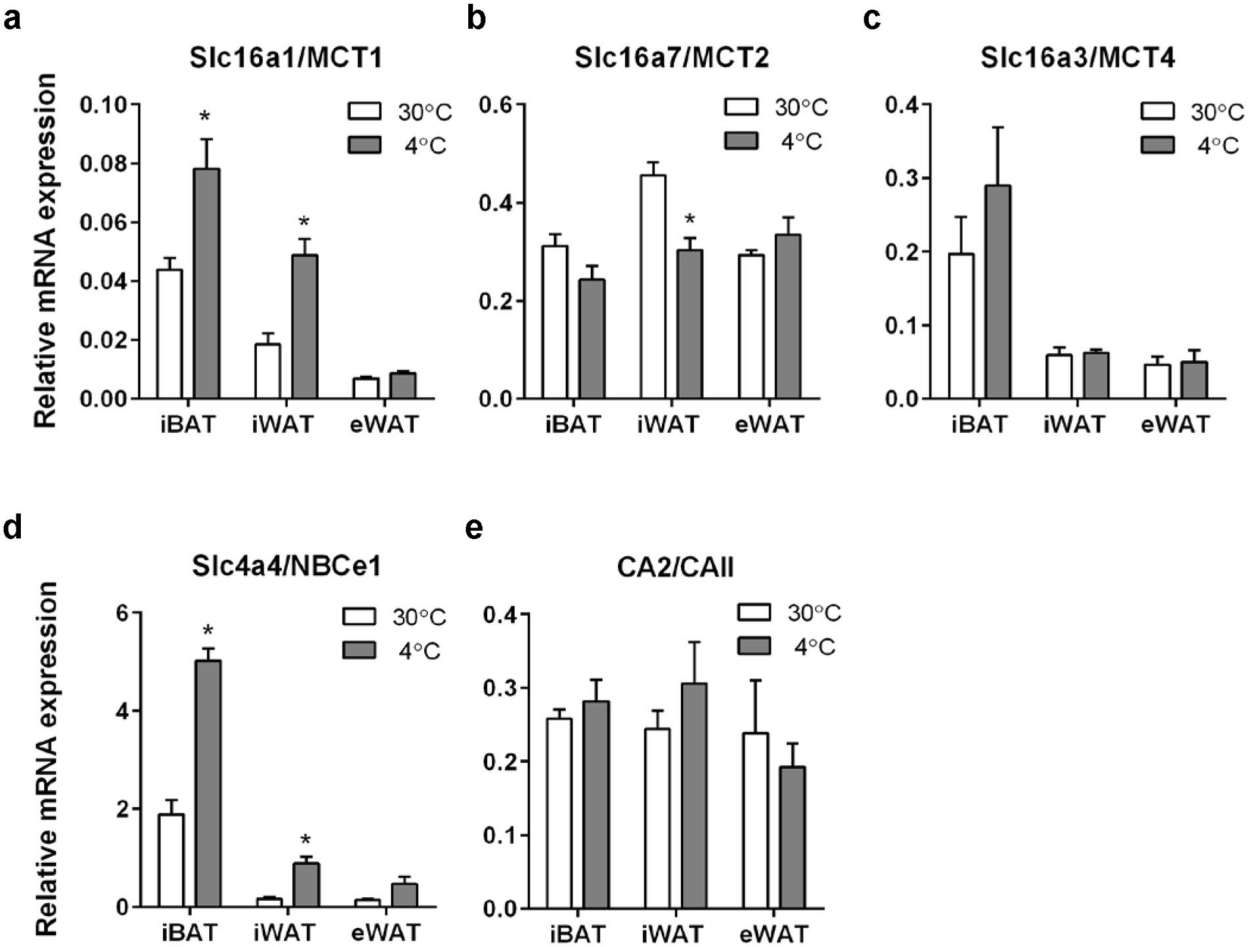
mRNA levels of acid-base transporters and enzymes in mouse iBAT, iWAT and eWAT are altered during cold-induced thermogenesis and browning. C57BL/6 mice were randomly divided into two groups and exposed to either 30 °C or 4 °C for 8 days. After 8 days, the mice were euthanized and iBAT, iWAT and eWAT frozen in liquid nitrogen and mRNA extracted, and qPCR analysis performed for MCT1, −2, −4, NBCe1, and carbonic anhydrase II (CAII) using TBP as a reference gene. Data are from 5 and 6 mice for 30 °C and 4 °C conditions, respectively. *p < 0.05, *t*-tests with multiple comparison correction.

We first assessed the relative expression levels of acid-base transporters in mouse iBAT, iWAT and eWAT and determined whether activation of BAT and induction of iWAT browning following cold exposure was associated with upregulation of acid-base transporters. Based on a qPCR screening of expression of major pH-regulatory transporters of the SLC4 (HCO_3_^−^ transporter), SLC9 (Na^+^/H^+^ exchanger), and SLC16 (MCT) families and carbonic anhydrases (CAs) (data not shown), we selected MCT1, −2, and −4, NBCe1, and CAII for further analysis. Upon induction by cold, thermogenic adipocyte markers were more strongly induced in iBAT and iWAT than in eWAT^26,27^. Mice were exposed to 30 °C or 4 °C for 8 days, and qPCR analysis was carried out on mRNA from iBAT, iWAT and eWAT, using TATA-binding protein (TBP) as reference gene^28^. At 30 °C, the relative mRNA levels of MCT1 was 0.04 in iBAT, 0.02 in iWAT, and less than 0.01 in eWAT. Upon cold induction, the relative MCT1 mRNA level approximately doubled in both iBAT and iWAT but was unaffected in eWAT. A similar pattern was seen for NBCe1, the relative expression of which more than doubled in iBAT and iWAT upon cold exposure (Fig. 1a,d). Relative MCT4 mRNA expression was 0.2 in iBAT and about 0.05 in iWAT and eWAT and was not significantly altered upon cold exposure (Fig. 1c). Finally, mRNA levels of MCT2 and CAII were similar in all three adipose tissue types and unaffected by cold exposure (Fig. 1b,e).

Collectively, these results show that MCT1 and NBCe1 mRNA levels are increased in iBAT and iWAT *in vivo* upon cold exposure.

### mRNA and protein levels of MCT1, MCT4 and NBCe1 are increased during adipocyte differentiation

**Figure 2:**
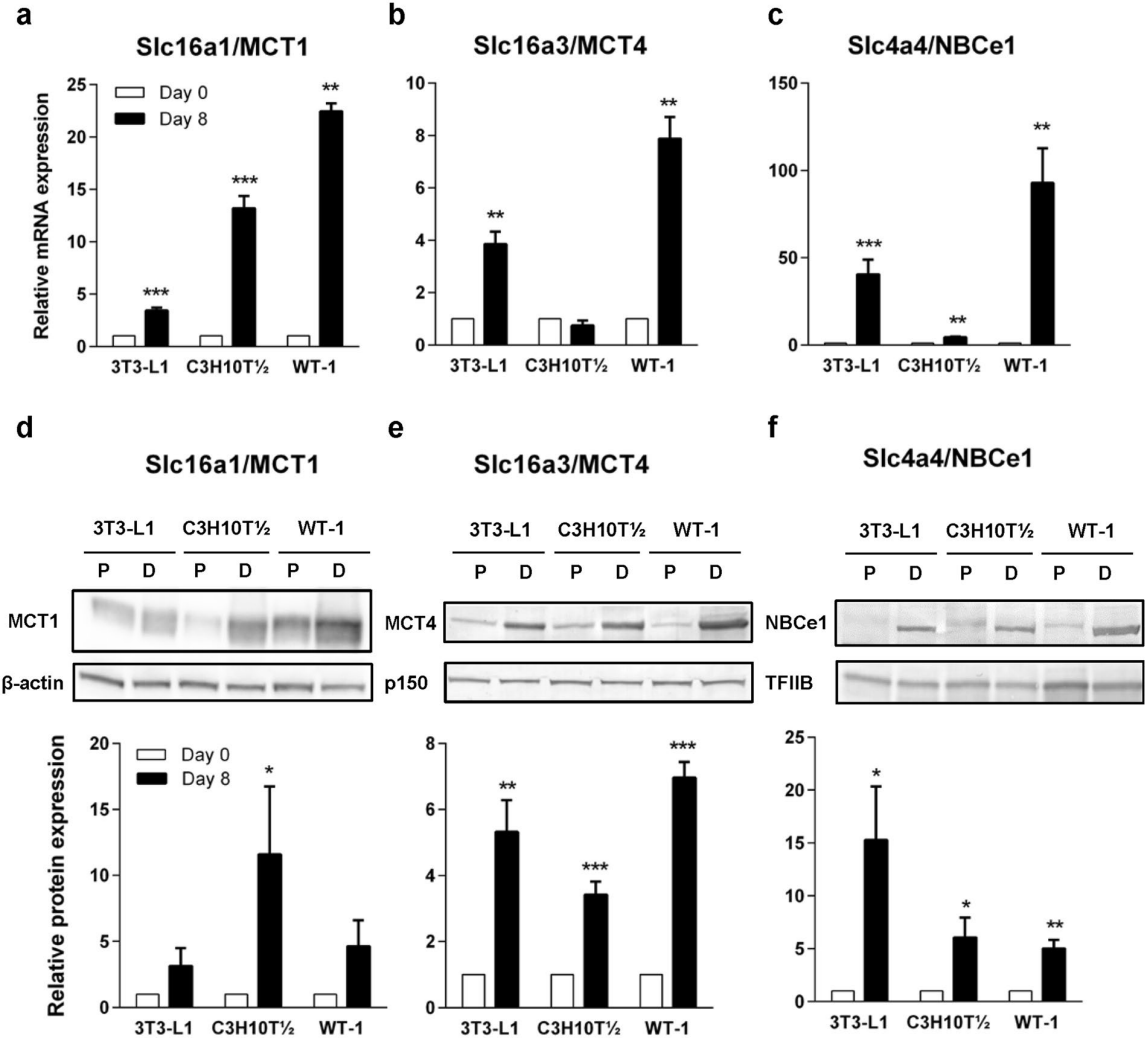
mRNA and protein levels of MCT1, MCT4, and NBCe1 are increased upon adipocyte differentiation. 3T3-L1, C3H10T½ and WT-1 pre-adipocytes were grown to confluency and either lysed and processed for mRNA extraction and qPCR analysis (**a**–**c**) or for SDS-PAGE and immunoblotting (**d**–**f**) at this point (day 0) or induced to differentiate into adipocytes as described in Materials and Methods, and harvested for qPCR analysis on day 8. TBP was used as a reference gene, and the Pfaffl method was applied to determine relative expression levels. The fold increase in mRNA level relative to that at day 0, of (**a**) MCT1, (**b**) MCT4, (**c**) NBCe1 is shown. Data are shown as mean with S.E.M. error bars, and represent 3 independent biological replicates for each gene. *, ** and ***: Significantly different from corresponding day 0 value, at p < 0.05, 0.01, and 0.001, respectively, paired two-tailed Students *t*-test. Panels d-f show the fold increase in protein level relative to that at day 0, of (**d**) MCT1, (**e**) MCT4, and (**f**) NBCe1. Representative blots are shown above each graph, with β-actin, p150^Glued^ and TFIIB as loading controls. Membranes were cut horizontally before blotting and edges slightly cropped for presentation. Graphed data are shown as mean with S.E.M. error bars, and represent 3–6 biological replicates for each cell type. P: pre-adipocyte, D: differentiated. ** and ** Significantly different from corresponding day 0 value, at p < 0.01 and 0.001, respectively, paired two-tailed Students *t*-test.

Next, we asked whether mRNA expression levels of essential pH regulatory proteins and enzymes are altered during white and brown adipocyte differentiation. 3T3-L1, C3H10T½ and WT-1 pre-adipocytes were differentiated for 8 days, and mRNA levels were evaluated before (designated day 0) and after (day 8) differentiation. The 3T3-L1 and C3H10T½ cell lines are white adipocyte models, whereas the WT-1 cell line is a brown adipocyte model. Oil Red O staining and immunoblotting for hormone-sensitive lipase (HSL) confirmed the robust differentiation using the protocol employed (Supplementary Fig. 1), in congruence with our previous reports^29,30^. Based on the *in vivo* expression levels and cold-induced changes (Fig. 1), we selected MCT1, MCT4, and NBCe1 for further analysis. Upon differentiation, the mRNA level of MCT1 increased significantly in all three adipocyte cell models (Fig. 2a) with increases from day 0 to day 8 of 4-fold in 3T3-L1, 13-fold in C3H10T½, and 22-fold in WT-1 adipocytes. The mRNA level of MCT4 was increased 4- and 8-fold in 3T3-L1 and WT-1 adipocytes, respectively, whereas there was no change in MCT4 expression upon differentiation of C3H10T½ cells (Fig. 2b). NBCe1 expression was increased about 40-fold in 3T3-L1, 4.5-fold in C3H10T½, and 90-fold in WT-1 cells upon differentiation (Fig. 2c). In contrast, the mRNA level of another major family member, NBCn1, was essentially unaltered upon differentiation in all cell lines studied (n = 3, data not shown). Immunoblotting analysis of cell lysates harvested before and after differentiation showed that the protein expression levels of MCT1 (Fig. 2d), MCT4 (Fig. 2e) and NBCe1 were likewise increased upon differentiation, qualitatively consistent with the mRNA data, although specific levels in the different cell lines varied.

These results show that MCT1, MCT4, and NBCe1 mRNA and protein levels are robustly increased upon adipocyte differentiation.

### Immunocytochemistry analysis confirms MCT1 and MCT4 upregulation during adipocyte differentiation

**Figure 3:**
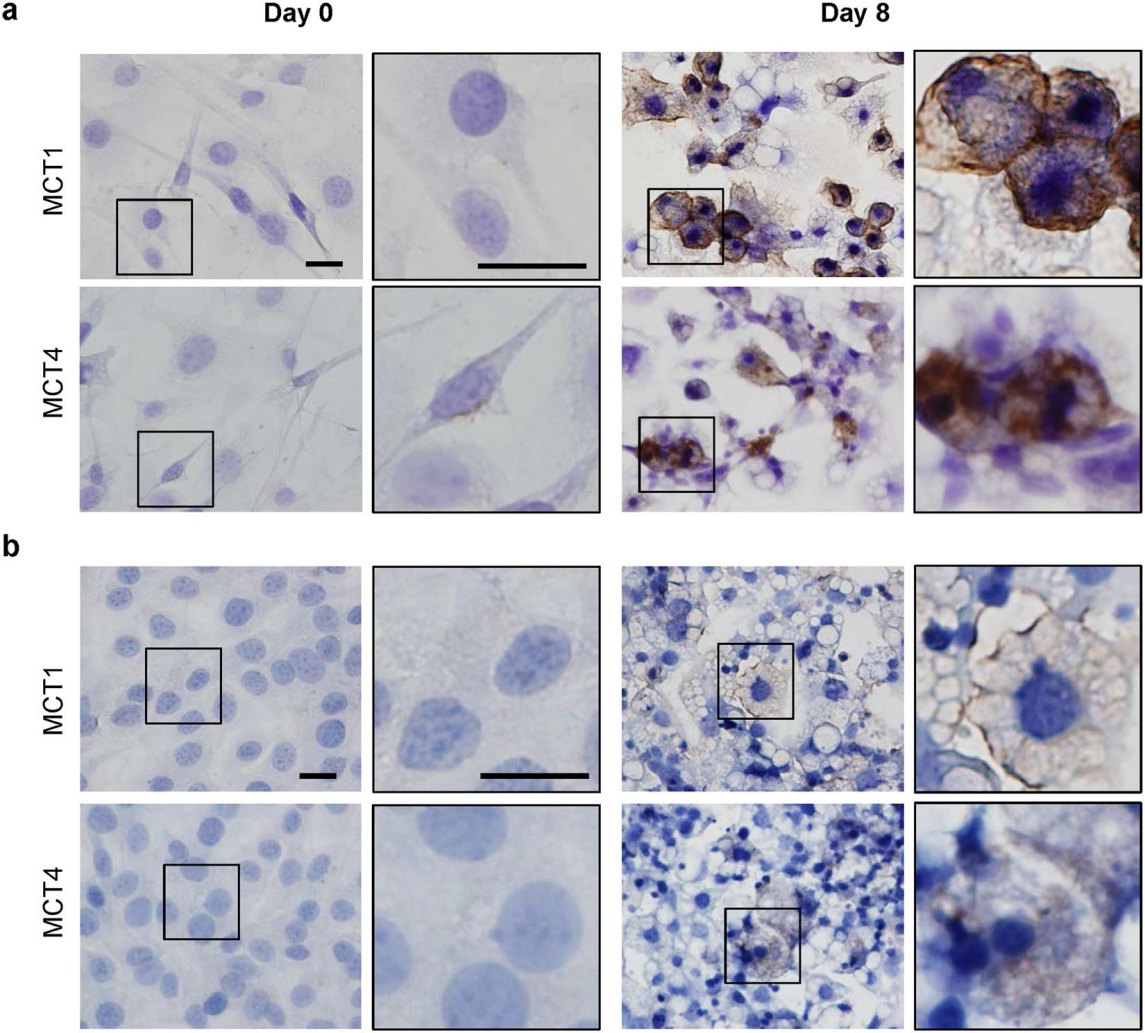
MCT1 and MCT4 localization in undifferentiated and differentiated 3T3-L1 and WT-1 adipocytes. 3T3-L1 (**a**) and WT-1 (**b**) pre-adipocytes were grown on glass coverslips, and either paraformaldehyde-fixed at this point (day 0), or differentiated to adipocytes as described in Materials and Methods, and fixed on day 8. Specimens were subjected to chromogenic staining with antibodies against MCT1 or MCT4 as indicated, and images were acquired at 40x magnification. Zoomed images are magnified from the regions marked with black boxes. Data are representative of 3 (3T3-L1) and 2 (WT-1) biological replicates. Scale bars: 20 µm for both.

Because of the marked upregulation of MCT1 and MCT4 observed in Fig. 2 and the recently proposed central role of lactate for adipocyte browning^17^, our further analyses focused on the lactate transporters. Immunocytochemistry analysis in 3T3-L1 and WT-1 pre-adipocytes and mature adipocytes confirmed the upregulation of both MCT1 and MCT4 during differentiation. In 3T3-L1 cells (Fig. 3a), MCT1 expression was essentially undetectable immunocytochemically in the pre-adipocytes, but strongly upregulated in the plasma membrane upon differentiation and appeared to predominantly localize to this compartment, although some intracellular staining was also detectable. MCT4 was also largely undetectable in pre-adipocytes and upregulated upon differentiation, yet exhibited stronger intracellular expression and substantial cell-to-cell variability of the expression level. The increased protein expression of MCT1 and MCT4 upon differentiation was also confirmed immunocytochemically in WT-1 cells, although the intensity of immunostaining was lower in this cell line (Fig. 3b).

### Lactate flux capacity is increased upon adipocyte differentiation, in a manner dependent on MCT activity

**Figure 4:**
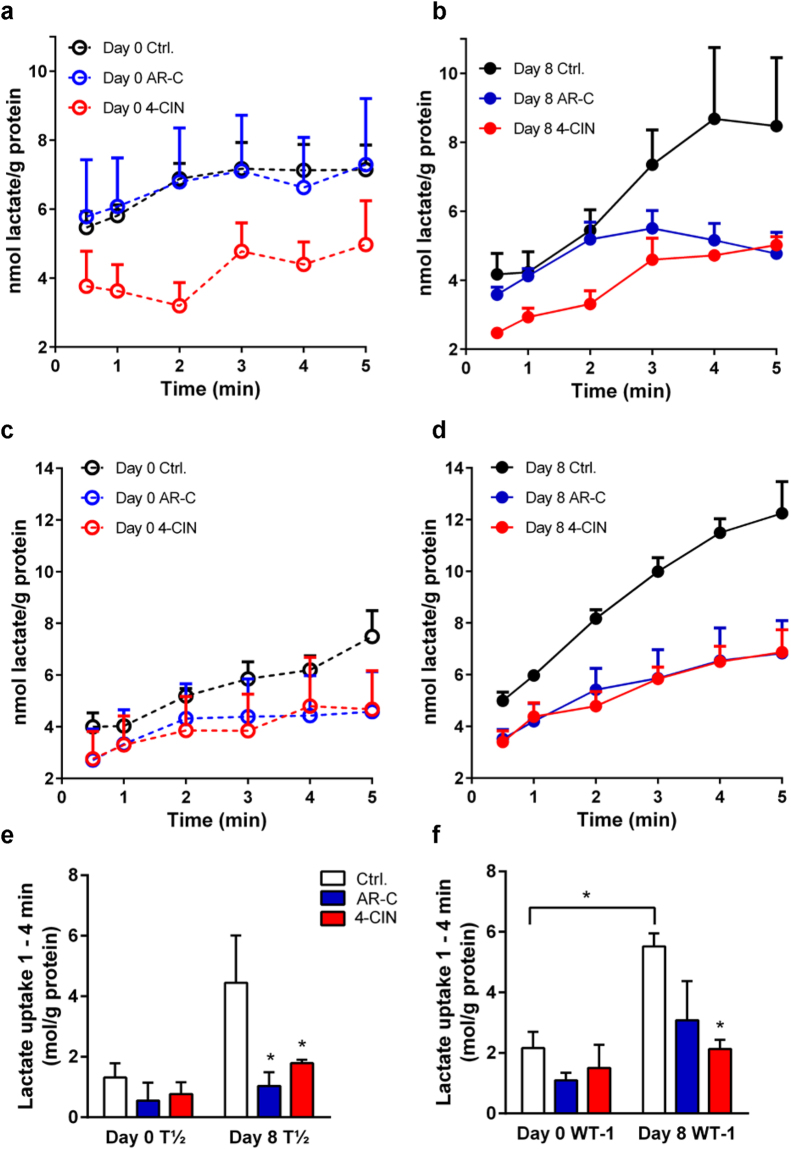
Lactate influx in undifferentiated and differentiated adipocytes. C3H10T½ and WT-1 cells were grown to confluency and either subjected to radioactive tracer analysis of lactate influx as described in Materials and Methods at this point (day 0), or induced to differentiate and lactate influx analysis was carried out on day 8. Panels a, b, and e show data from C3H10T½ cells, panels c, d, and f show data from WT-1 cells. Where indicated, AR-C155858 (10 µM, from 1 h prior to start of influx) or 4-CIN (5 mM, from 15 min prior to starting of influx) was present to inhibit MCT1/−2 or all MCTs, respectively. Data are shown either as the influx over time in nmoles/mg protein (panel a–d), or as the net influx from time 1 to 4 min (**e**, **f**; mean with S.E.M. error bars), and represent 3 independent biological replicates for each condition. *p < 0.05, two-way ANOVA with Dunnett’s post-test (Ctrl. vs. AR-C and 4-CIN) or Students *t*-test (day 0 vs day 8).

We next asked whether the upregulation of MCT1 and MCT4 upon differentiation translates into increased lactate flux capacity. Lactate influx measurements were carried out in C3H10T½ and WT-1 cells, representative of white and brown adipocytes, respectively. Importantly, MCTs facilitate lactate transport in either direction, the relation between the influx and efflux kinetics being defined by the Haldane equation (*V*_max_/*K*_m_)[influx] = (*V*_max_/*K*_m_)[efflux], and the *direction* of the flux solely being dictated by the driving force for transport, i.e., the prevalent lactate and H^+^ gradients^18^. Hence, the influx measured here is simply an expression of the capacity for flux in either direction and under physiological conditions, thermodynamics is likely to dictate outward-directed lactate-H^+^ transport via the MCTs in the adipocytes (see Discussion). To assess the contributions from the MCTs, we employed the broad MCT inhibitor 4-CIN, and the MCT1/−2 specific inhibitor AR-C55858 (AR-C). In C3H10T½ cells, lactate influx increased upon differentiation and was inhibited to a similar extent in the presence of either AR-C or 4-CIN (Fig. 4a–b,e). A similar pattern was seen in WT-1 cells (Fig. 4c–d,f). Also in these cells, AR-C and 4-CIN reduced the flux, although only statistically significant for 4-CIN.

**Figure 5:**
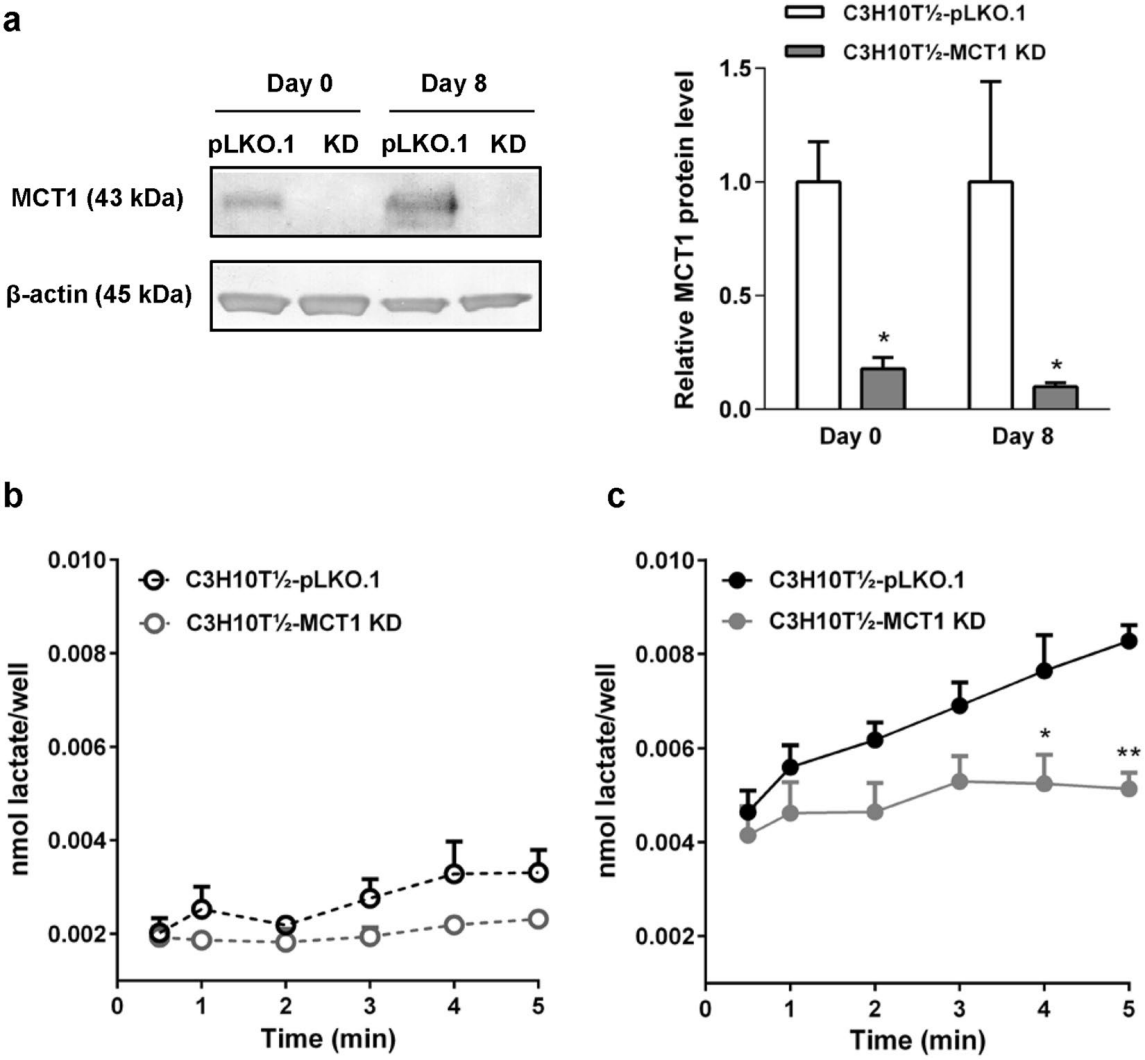
Lactate influx in C3H10T½ cells is attenuated by stable knockdown of MCT1. MCT1 was stably knocked down in C3H10T½ cells by lentiviral shRNA transduction as described in Materials and Methods. (**a**) Relative MCT1 protein levels in C3H10T½ cells expressing empty control plasmid (pLKO.1) or MCT1 shRNA (KD), at day 0 (undifferentiated) and day 8 after differentiation induction. β-actin is shown as loading control. Membranes were cut horizontally before blotting and edges slightly cropped for presentation. The bar chart shows mean data from 3 independent experiments, normalized to control values at day 0 and day 8. (**b**) Tracer measurements of lactate influx were carried out as described in the legend to Fig. 4, and net influx calculated as nmol lactate ·well^−1^ before (**b**) and after (**c**) differentiation. The figure shows means with SEM error bars of 3 independent biological replicates for pLKO.1 (black symbols) and MCT1 KD (gray symbols) cells. *, **p < 0.01, Two-way ANOVA. Using linear regression of the data in b and c to calculate the flux, and correcting for the fact that MCT1 KD reduced the growth of the cells, resulting in approximately 30% lower protein levels, average fluxes of 0.67 nmol lactate · g protein^−1^ · min^−1^ in undifferentiated pLKO.1 control cells, 0.32 nmol lactate · g protein^−1^ · min^−1^ in undifferentiated MCT1 KD cells, 1.41 nmol lactate · g protein^−1^ · min^−1^ in differentiated pLKO.1 cells, and 0.67 nmol lactate · g protein^−1^ · min^−1^ in differentiated MCT1 KD cells can be calculated.

The similar effect of the broad MCT inhibitor 4-CIN and the MCT1/−2 specific AR-C on the lactate flux suggested that MCT1 and/or −2 were predominantly responsible for the flux, although MCT4 is also upregulated. To more specifically assess the contribution of MCT1 to the flux, we carried out stable lentiviral knockdown of this transporter in C3H10T½ cells (in which both MCT1 and MCT4 protein levels are significantly upregulated upon differentiation, see Fig. 2) and assessed the lactate influx capacity. After knockdown, the protein level of MCT1 in differentiated C3H10T½ cells was reduced to about 15% of that in pLKO.1 empty controls (Fig. 5a). MCT1 knockdown had no effect on lactate flux in undifferentiated C3H10T½ cells (Fig. 5b), yet markedly reduced the flux in differentiated cells (Fig. 5c). Using linear regression of the data in Fig. 5b and c to calculate the flux, and correcting for the fact that MCT1 KD somewhat slowed cell growth, resulting in approximately 30% lower protein levels, the average lactate flux was determined at 0.67 nmol lactate · g protein^−1^ · min^−1^ in undifferentiated pLKO.1 control cells, 0.32 nmol lactate · g protein^−1^ · min^−1^ in undifferentiated MCT1 KD cells, 1.41 nmol lactate · g protein^−1^ · min^−1^ in differentiated pLKO.1 cells, and 0.67 nmol lactate · g protein^−1^ · min^−1^ in differentiated MCT1 KD cells (n = 3 for all conditions). To distinguish between effects of loss of MCT1 function on adipocyte differentiation, and effects directly on the flux in the differentiated cells, we performed the full differentiation of C3H10T½, 3T3-L1, and WT-1 cells in absence or presence of AR-C (Supplementary Fig. 1), and validated differentiation by Oil Red O staining and immunoblotting for HSL, MCT1 and MCT4. As seen, the inhibitor had no detectable effect on the differentiation *per se*, nor on the expression levels of MCT1 and −4, in any of the three cell types.

Taken together, these results show that lactate transport capacity is increased upon adipocyte differentiation and that MCT1 appears to be responsible for a substantial fraction of the lactate influx capacity of differentiated adipocytes.

### Inhibition of lactate transporters alters the metabolic phenotype of WT-1 cells

**Figure 6:**
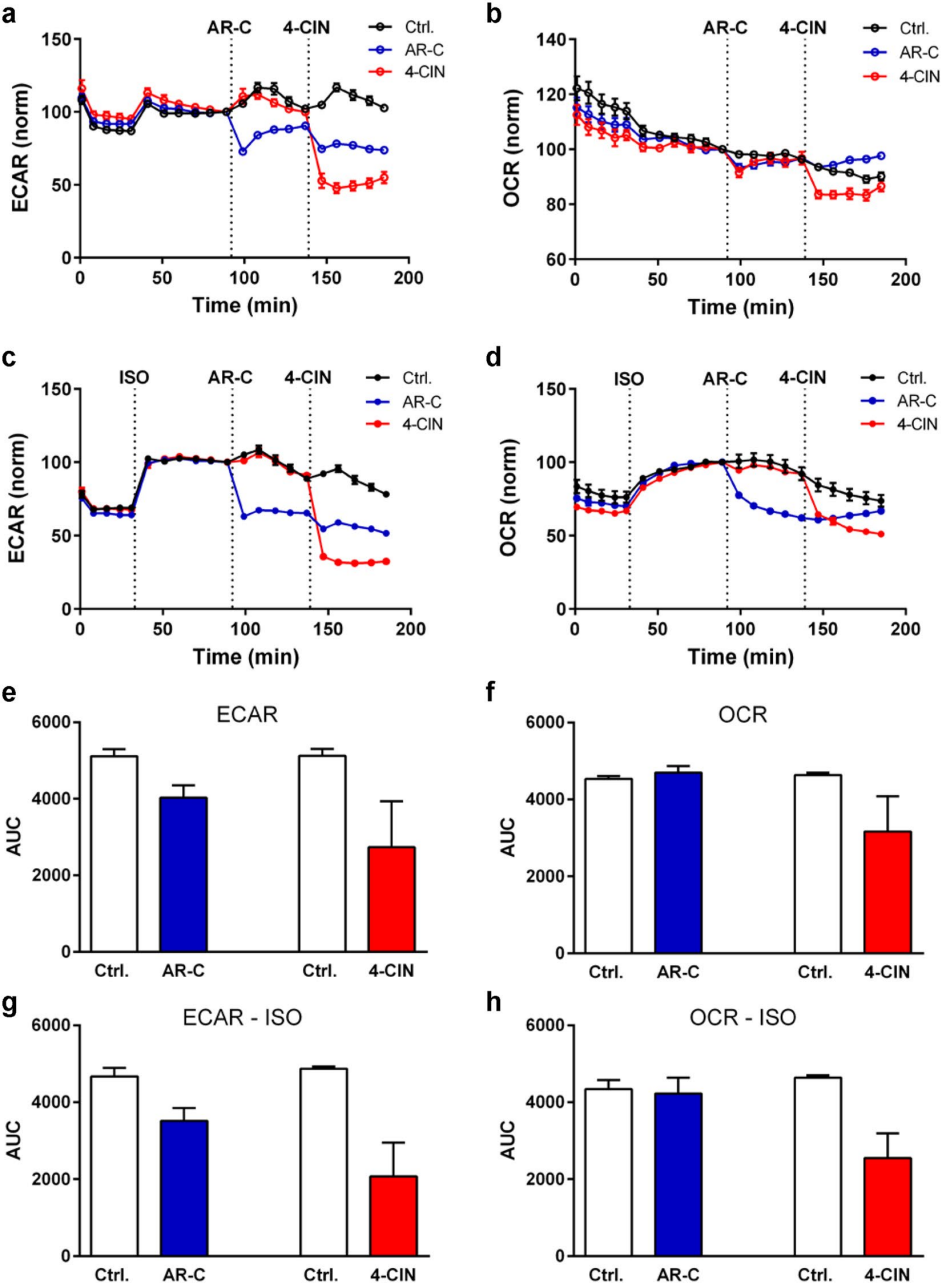
Effect of MCT inhibition on OCR and ECAR of differentiated WT-1 adipocytes under control conditions and after isoproterenol stimulation. On day 6 after induction of differentiation, WT-1 adipocytes were replated into gelatin-coated XF96 Seahorse Analyzer microplates. Analysis of OCR and ECAR was carried out on differentiation day 8 as described in Materials and Methods. Experiments were carried out under control conditions (**a**,**b**,**e**,**f**) or in the presence of isoproterenol (1 µM, injected as indicated by the dotted line) as a β-adrenergic agonist (**c**,**d**,**g**,**h**). AR-C (10 µM) and 4-CIN (5 mM) were injected at the times indicated. The data shown in a-d is representative of 3 independent experiments and is given as mean with SD error bars, of 15-16 replicates per condition. **e**–**g**. Data summarized over all 3 Seahorse experiments (each performed in 15-16 replicates) are shown as mean with SEM error bars. For each well in each experiment, data from all measurements was normalised to the last point before inhibitor injection and the area under the curve (AUC) was determined for the time-course after that injection.

We finally examined whether inhibition of MCT activity affected the metabolic activity of differentiated adipocytes. Brown adipocytes, i.e. WT-1 cells, were employed to allow us to assess the effect of MCT inhibition on the robust β-adrenergically stimulated metabolic activity of these cells. Cells were grown to confluence, induced to differentiate, and on day 6 replated into Seahorse XF Analyzer plates for measurements of OCR and ECAR at day 8. OCR and ECAR were measured under control conditions and in presence of AR-C or 4-CIN to assess the impact of MCT inhibition. Data are shown as a representative experiment (Fig. 6a–d) and data summarized over 3 independent experiments (Fig. 6e–h). It may be noted that between-experiment variability of absolute values is large because of variability in the degree of differentiation. Hence, panel e-f does not reach statistical significance, but the qualitative effects are similar for all three experiments. AR-C attenuated ECAR, but had no detectable effect on OCR. A similar but slightly larger effect on ECAR was seen for 4-CIN, with a tendency for inhibition also of OCR (Fig. 6a–b,e–f).

Cold-induced noradrenaline release stimulates thermogenesis in BAT as well as in browning-prone white adipocytes^9^. To determine whether β-adrenergic stimulation altered the MCT dependence of OCR and ECAR, the β-adrenergic agonist isoproterenol (ISO) was added, followed by addition of AR-C or 4-CIN as above. As expected, isoproterenol treatment increased both OCR and ECAR, and under these conditions, ECAR was strongly attenuated by both AR-C and 4-CIN, and OCR by 4-CIN (Fig. 6c,d,g,h). The effects of AR-C and 4-CIN on ECAR and OCR do not reflect altered β-adrenergic signaling, as Ser133-phosphorylation of cAMP-responsive element binding protein (CREB) and Ser660-phosphorylation of HSL were unaffected by the inhibitors (Supplementary Figure 2a,b). Moreover, AR-C and 4-CIN did not increase the activity of p38 MAPK or the cleavage of poly-ADP-ribose (PARP), suggesting that the treatments were not associated with nonspecific toxicity (Supplementary Figure 2c).

These results indicate that MCTs contribute to net acid extrusion from WT-1 brown adipocytes under basal and β-adrenergically stimulated conditions and that MCT activity facilitates β-adrenergically induced oxidative phosphorylation in these cells.

## Discussion

Metabolically active cells are dependent on high rates of lactate and H^+^ extrusion to maintain their cytosolic pH (pH_i_) and therefore their glycolytic flux, which is exquisitely sensitive to pH_i_^24,31^. Lactate flux across the plasma membrane is mediated by the SLC16/MCT family of lactate-H^+^ cotransporters, while extrusion of acid equivalents additionally involves Na^+^/H^+^ exchangers and Na^+^,HCO_3_^−^ cotransporters^25,32,33^. Adipocytes contribute significantly to systemic lactate homeostasis, with important physiological and pathophysiological implications^6,10–12^. Under normal physiological conditions, adipocytes may contribute with lactate for extra-adipose metabolism during fasting, e.g. providing lactate for hepatic gluconeogenesis. Pathophysiologically, the plasma lactate level is increased in obese and diabetic patients compared to lean controls, reflecting increased adipocyte mass and increased glucose conversion to lactate with increasing adipocyte size^10^. In addition, increased plasma lactate levels correlate positively with insulin resistance^10^.

In this study, we therefore asked whether lactate transporters and other essential regulators of cellular acid/base homeostasis are upregulated during cold-induced thermogenesis and browning, and upon adipocyte differentiation. The key findings of this work are that the monocarboxylate transporter MCT1 and the Na^+^, HCO_3_^−^ cotransporter NBCe1 are upregulated in mouse iBAT and iWAT upon cold exposure, that MCT1 and MCT4, as well as NBCe1, are upregulated during both white and brown adipocyte differentiation, accompanied by increased, predominantly MCT1-driven lactate flux capacity, and that MCT inhibition reduces brown adipocyte ECAR, and after β-adrenergic stimulation also OCR.

Previous data on MCT expression in adipocytes is very limited and to some extent contradictory^17,21–23^. MCT1 and MCT4 protein expression were detected in cultured human adipocytes^23^. MCT1 protein expression was demonstrated in rat WAT along with 4-CIN-inhibitable MCT activity, whereas MCT4 was not detected^21^; and in mice, MCT1 was found in BAT, but not in WAT^22^. In the present work, we show unequivocally, in three different adipocyte cell lines, that MCT1 and MCT4 expression is increased upon adipocyte differentiation.

The upregulation of MCT1 and MCT4 is in accordance with the increased lactate production in adipocytes upon differentiation^34^ and the resulting increase in requirement for lactate efflux. Our finding that MCT1 expression in iWAT is upregulated by cold exposure *in vivo* agrees well with previous findings that mitochondrial uncoupling in WAT increases lactate output while reducing both fatty acid synthesis and lipogenesis^35^. Also in congruence with our data, MCT1 expression in brown adipose tissue was previously found to be most prominent in fetal, newborn, and cold-exposed mice^22^. This led to the suggestion that MCT1 might contribute to lactate uptake as an energy source for thermogenesis^22^. MCTs can in principle transport equally well in both directions^18^, the direction of transport being solely determined by thermodynamics. It should be kept in mind, however, that the driving force for MCT in cells with a high glycolytic acid- and lactate production, such as activated adipocytes, is more likely to be in the outward direction, consistent with reports of lactate release from WAT *in vivo* ^10–12^ and lactate production during browning^17^ Hence, we favor the interpretation that high MCT1 expression in BAT reflects the high lactate production in these highly metabolically active cells and is important for the lactate efflux required to maintain their normal metabolism^35^. MCT4 expression, while not significantly higher after cold exposure *in vivo*, was higher in BAT than in iWAT and eWAT *in vivo*, and its differentiation-induced increase was higher in the BAT-like WT-1 cells than in 3T3-L1 and CH310T½ cells. Thus, our findings suggest that this transporter may also be important for BAT function.

In agreement with the upregulation of MCT expression, lactate flux capacity was robustly increased upon adipocyte differentiation. AR-C is an inhibitor of both MCT1 and −2. Although MCT2 mRNA expression was reported by non-quantitative PCR in human adipocytes^23^, MCT2 protein expression has, to our knowledge, not been reported in adipocytes. Collectively, the attenuation of lactate uptake by broad inhibition of MCTs (4-CIN), by selective inhibition of MCT1/−2, and by stable knockdown of MCT1 point to a major role of MCT1 in the adipocyte plasma membrane lactate flux capacity, although we cannot rule out contributions from MCT2 and/or MCT4.

In addition to inhibiting lactate efflux, AR-C and 4-CIN also acutely inhibited ECAR in differentiated brown adipocytes. This most likely reflects a combination of inhibition of glycolysis (and hence both general metabolism and downstream *de novo* lipogenesis) and UCP-1 function (see below) as a consequence of intracellular lactate- and acid accumulation. Cold-induced noradrenaline release rapidly stimulates thermogenesis in BAT as well as in brown-like adipocytes^9^. Remarkably, the isoproterenol-induced stimulation of brown adipocyte metabolism (OCR, ECAR) in the present work was strongly and rapidly inhibited by inhibition of MCT1/−2, and to a slightly greater extent by broad inhibition of MCTs by 4-CIN. Although 4-CIN is also an inhibitor of the mitochondrial pyruvate carrier (MPC)^18^, the near-instantaneous effect of 4-CIN in the present study indicates that the effect of 4-CIN is not primarily an effect on MPC.

While this work clearly demonstrates a major role of MCT-mediated lactate transport in adipocyte metabolism, the MCT-mediated changes in adipocyte lactate content may serve additional roles. The conversion of pyruvate to lactate is important for NAD^+^ regeneration and hence maintenance of metabolic flux and, as discussed above, lactate released from adipocytes appears to be a major fuel for e.g. hepatic metabolism. However, lactate is increasingly recognized to be not solely a metabolite, but also a signaling molecule^19^. A recent study demonstrated that lactate, in a redox-dependent signaling process, induced adipocyte browning, in a manner proposed to involve apparently differential roles of MCT1 and MCT4^17^. Curiously, in that work, UCP1 induction was abrogated by unspecific pharmacological inhibition of MCTs, but augmented by knockdown of MCT4 but not of MCT1^17^. This was suggested to indicate a predominant role for intracellular lactate in the browning process^17^, and while such a role seems plausible, the complex effects of MCT manipulation, suggest that the role(s) of the lactate transporters is still incompletely understood. Our results raise the important possibility that adipocyte MCTs may have a therapeutic relevance in the context of obesity. Metabolic disturbances such as obesity and diabetes are associated with changes in WAT-related lactate homeostasis^6,10–12^. Interestingly, a haploinsufficient MCT1 mouse exhibits resistance to diet-induced obesity due to decreased fat accumulation in WAT and liver, the former reflecting reduced adipocyte size^36^. While MCT1 activity in both brain and gut probably contribute to this phenotype, it seems likely that reduced WAT-endogenous MCT1 function may also contribute. Unfortunately, due to the partial knockout model, MCT1 expression in BAT was essentially unaffected, hence the possible impact of this tissue could not be evaluated.

Importantly, glycolysis is not the only metabolic process contributing to cellular acid production (and hence to ECAR)^25,33^. Metabolic CO_2_ production resulting from oxidative phosphorylation is a major contributor to acid production as CO_2_ is converted to H^+^ and HCO_3_^−^, facilitated by carbonic anhydrase activity. In accordance with this, adipocyte differentiation was associated with strongly increased expression of the Na^+^,HCO_3_^−^ cotransporter NBCe1. Several functional roles can be envisaged for the high expression of NBCe1 in differentiated adipocytes: Firstly, the above-mentioned major inhibitory effect of acidic pH_i_ on PFK-1^24^ and the inhibitory effect of acidic pH_i_ on UCP1 function^37,38^ suggest that adipocytes likely require increased capacity for net acid extrusion upon differentiation. Second, HCO_3_^−^ is important for adipocyte function beyond its role in pH_i_ regulation, because of its involvement as a substrate in *de novo* lipogenesis (in the mitochondrial production of oxaloacetate to form citrate, and in the cytosolic formation of malonyl-CoA from acetyl-CoA), in a process is dependent on CAII^39^. We propose that NBCe1 may play a dual role in adipocytes, as a pH_i_ regulator and provider of substrate for HCO_3_^−^ dependent metabolic processes. While this by no means excludes that these transporters might also play important roles during adipocyte differentiation, it is interesting to note that two other major pH regulatory transporters, NHE1 and NBCn1, were not significantly upregulated at the mRNA level during differentiation in any of the adipocyte models tested. The literature on pH_i_ regulation in adipocytes is sparse and contradictory. Insulin was reported to induce either Na^+^/H^+^ exchange-dependent^40^ or Na^+^/H^+^ exchange-independent^41^ alkalinization in rat adipocytes. Na^+^/H^+^ exchange was found to be stimulated by insulin in 3T3-L1 pre-adipocytes but not in mature 3T3-L1 adipocytes^42^. While future studies should assess the precise roles of HCO_3_^−^ transport in adipocyte pH_i_ regulation, our work shows that differentiated adipocytes selectively upregulate transporters and enzymes important for HCO_3_^−^ transport.

*In conclusion*, this is the first report to show that MCT1 and MCT4, as well as NBCe1, are strongly upregulated at the mRNA and protein level upon adipocyte differentiation. Differentiation was accompanied by enhanced lactate flux capacity, which was strongly reduced by MCT inhibition and MCT1 knockdown. In differentiated brown adipocytes glycolysis (ECAR), and after noradrenergic stimulation also oxidative metabolism (OCR), was abolished by MCT inhibition. Finally, MCT1 and NBCe1 were upregulated in iWAT *in vivo* upon cold induction. We propose that upregulation of MCT1- and MCT4-mediated lactate flux capacity and NBCe1-mediated HCO_3_^−^/pH_i_ homeostasis is of central importance for the physiological function of differentiated adipocytes.

## Materials and Methods

### Reagents and antibodies

AR-C 155858 (AR-C) was from AdooQ Bioscience, α-Cyano-4-hydroxycinnamic acid (4-CIN) and isoproterenol from Sigma-Aldrich, and^14^C-lactate from PerkinElmer. Antibodies against MCT1 and MCT4 were from Millipore, and antibodies against carbonic anhydrase II (CAII) and NBCe1 from Abcam. Antibodies against p150^Glued^ and transcription factor IIB (TFIIB) were from BD Biosciences and Santa Cruz Biotechnology, respectively. Unless otherwise mentioned, other reagents were purchased from Sigma-Aldrich. Antibodies against hormone-sensitive lipase (HSL), phospho-Ser660-HSL, phospho-Ser133 cAMP-responsive element binding protein (CREB), poly-ADP-ribose (PARP), and phospho-Thr180/Tyr182-p38 MAPK were from Cell Signaling Technology, Leiden, The Netherlands.

### Cell culture and differentiation

3T3-L1 cells^43^ and C3H10T½ cells^44^ were kindly provided by Dr. Karsten Kristiansen. The WT-1 cell line established by immortalization of brown pre-adipocytes from newborn mice with SV40 large T antigen was kindly provided by Dr. C. Ronald Kahn^45^. 3T3-L1, C3H10T1/2 and WT-1 cells were propagated in DMEM (Invitrogen) containing 35 mM HCO_3_^−^, 0.008 g/L biotin, 0.008 g/L D-pantothenic acid, hemicalcium salt, 10% Fetal Bovine Serum (FBS) (calf serum for 3T3-L1 until day 0), and 1% penicillin/streptomycin mix (hereafter complete DMEM) at 37 °C/5% CO_2_. The medium was replaced every 2-3 days, and cells were passaged by gentle trypsinization when reaching 70–80% confluency. For differentiation, cells were grown to confluency in DMEM, and 1-2 day post-confluent cells (designated day 0) were induced to differentiate by adding DMEM containing 1 μM dexamethasone, 0.5 mM methyl isobutyl xanthine (MIX), 5 μg/ml insulin/transferrin/selenium (I/T/S) solution (Gibco) and 0.5 μM rosiglitazone (Cayman Chemical). For cells used in immunoblot analysis, medium was refreshed with complete DMEM supplemented with 5 μg/ml I/T/S and 0.5 μM rosiglitazone on day 2, and with complete DMEM containing 0.5 μM rosiglitazone on days 4 and 6. For all other experiments, medium was refreshed with complete DMEM containing 5 μg/ml I/T/S and 0.5 μM rosiglitazone on day 2, and with complete DMEM on days 4 and 6.

### Oil Red O staining

To confirm differentiation, cells were stained with Oil Red O. Following treatments as indicated, the cells were washed twice with phosphate-buffered saline (PBS) and fixed in 3.7% formaldehyde for 1 h. After aspiration of the formaldehyde, the cells were stained with Oil Red O for 1 h. Oil Red O was prepared by dissolving 0.5 g Oil Red O (Sigma-Aldrich) in 100 ml 2-propanol and diluting it with water (6:4), followed by filtration. Stained cells were washed carefully in PBS and covered with water until photographed using the 20X lens of a Leica DMI 6000 microscope.

### Mouse cold exposure experiments

Mice were single-caged 10 weeks old male C57BL/6 mice (Taconic), housed at room temperature and fed standard chow diet. Mice were transferred to 4 °C or 30 °C for 8 days, after which they were killed by cervical dislocation. iBAT, iWAT, and epididymal WAT (eWAT) were excised, frozen in liquid nitrogen and stored at −80 °C.

The mouse experiments were approved by the Danish Animal Research Authority (Dyreforsøgstilsynet) and conducted in accordance with Danish legislation.

### mRNA analysis and quantitative real-time PCR

For Fig. 1, isolation of total RNA, reverse transcription and quantitative real-time PCR was performed as described^29^, except that the SensiFAST SYBR Lo-ROX Kit (Bioline) was used. For Fig. 2, isolation of total RNA from cultured cells was performed using *NucleoSpin® RNA II* (Macherey-Nagel, Germany), according to the manufacturer’s instructions. Total RNA was reverse-transcribed using Superscript III Reverse Transcriptase (Invitrogen, Carlsbad, CA). cDNA transcripts were amplified by qPCR using SYBR Green (Applied Biosystems, Cheshire, UK). qPCR analysis was carried out in 384 well format using an ABI7900 qPCR machine and the protocol [96 °C for 30 s; 55 °C for 1 min; 72 °C for 30 s] × 40. TATA-binding protein (TBP) was used as a reference gene, and the Pfaffl method was employed for determining relative expression levels. Primer sequences were:

NBCe1 fw: GGTGTGCAGTTCATGGATCGTC, NBCe1 rv: GTCGCTATCCAAACTTCCTTTC; NBCn1 fw: GCAAGAAACATTCTGACCCTCA, NBCn1 rv: GCTTCCACCACTTCCATTACCT; MCT1 fw: TGTTGTTGCAAATGGAGTGT, MCT1 rv; AAGTCGATAATTGATGCCCATGCCAA; MCT4 fw: TATCCAGATCTACCTCACCAC, MCT4 rv: GGCCTGGCAAAGATGTCGATGA; CAII fw: CAAGCACAACGGACCAGA, CAII rv: ATGAGCAGAGGCTGTAGG; CAIII fw: GCTCTGCTAAGACCATCC, CAIII rv: ATTGGCGAAGTCGGTAGG; NHE1 fw: TCTCCCTCTGGATCCTTCTGGC, NHE1 rv: GCCACCACGAAGAAGCTCAGGA; TBP fw: ACCCTTCACCAATGACTCCTATG; TBP rv: ATGATGACTGCAGCAAATCGC.

### Immunoblotting

Cells were grown to confluence in 6-well plates. Twoday post-confluent cells were either harvested (undifferentiated cells, day 0) or differentiated, followed by harvest for immunoblotting at day 8. Immunoblotting was carried out essentially as previously described^46^. Briefly, cells were washed in ice-cold PBS and lysed in warm lysis buffer (1% sodium dodecyl sulfate (SDS), 10 mM Tris-HCl, 1 mM NaVO_3_, pH 7.5). Lysates were sonicated, and debris pelleted by centrifugation (5 min, 20,000 g, 4 °C). Protein content was determined (DC Protein Assay kit, Bio-Rad), equalized for all samples by ddH_2_O addition, and NuPAGE LDS 4x Sample Buffer (Invitrogen) and dithiothreitol were added. Proteins were separated by denaturing and reducing SDS-PAGE using pre-cast NuPAGE 10% Bis-Tris gels (Life Technologies), NuPAGE MOPS SDS Running Buffer (Life Technologies), and Benchmark protein ladder. Separated proteins were transferred to a nitrocellulose membrane (Invitrogen) using NuPAGE Transfer Buffer (Life Technologies). Membranes were stained with Ponceau S, blocked in blocking buffer (5% nonfat dry milk in Tris-buffered saline plus 0.1% Tween 20 (TBST)) for 1 h at 37 °C, incubated with primary antibodies overnight at 4 °C, washed in TBST, and either incubated with alkaline phosphatase-conjugated secondary antibodies for 1–2 h, washed in TBST, and developed using BCIP/NBT Phosphatase Substrate (KPL), or incubated with Horseradish Peroxidase (HRP)-conjugated secondary antibodies, washed in TBST, and developed using Pierce ECL Western Blotting Substrate (Thermo Scientific). Membranes were scanned, and band intensity was quantified densitometrically using UN-SCAN-IT 6.1 (Silk Scientific).

### Immunocytochemistry

Cells were grown on glass coverslips. One day post-confluent cells (day 0) were either washed in ice-cold PBS, fixed in 3.7% paraformaldehyde, and washed extensively in PBS, or induced to differentiate as above, followed by fixation on day 8. Coverslips were stored at −20 °C until use. For immunocytochemistry, cells were thawed at room temperature (RT) and washed 5 min in TBS followed by incubation with TBS, 0.5% Triton X-100 for 10 min and washed in TBS 2 × 5 min. Immunocytochemistry was performed using the EnVision FLEX High pH Kit (Dako, Denmark). Samples were blocked for 20 min in EnVision FLEX Peroxidase-Blocking Reagent (Dako) and incubated with primary antibody for 60 min at RT. The antibodies were diluted in Antibody Diluent (Dako) at 1:100 (MCT1) or 1:200 (MCT4). Next, samples were incubated with EnVision FLEX/HRP solution and stained with EnVision FLEX DAB + Chromogen (Dako). Between incubations, the samples were washed with EnVision FLEX washing buffer. Lastly, cells were counterstained with Mayer’s hematoxylin.

### Stable lentiviral knockdown

HEK293T cells were propagated in DMEM containing 5% FBS and 1% penicillin/streptomycin (complete DMEM). For retrovirus packaging, HEK293T cells were seeded in 6 cm dishes. At 70% confluence, full growth medium was replaced by DMEM without penicillin/streptomycin. Next, 2.75 μg lentiviral vector (pLKO-siMCT1, pLKO.1 empty vector, Sigma-Aldrich), 0.75 μg of the two packaging plasmids (pRSV-Rev, pMDLg/pRRE, Addgene plasmids # 12253, 12251) and 0.75 μg envelope plasmid (pMD2.G, Addgene plasmid # 12259) (kindly provided by Dr. Didier Trono), were mixed with 12.5 μL FuGENE and DMEM to a total volume of 250 μL, incubated 15 min at RT and added to the cells. Next day, medium was replaced by complete DMEM. 3T3-L1 and C3H10T½ cells were seeded in 6 cm dishes to ~20% confluence, medium was removed and 1.5 mL complete DMEM and 4.5 μL polybrene (5 µg/ml) were added to each dish. The HEK293T cell medium containing the shRNA lentiviral particles was sterile filtered and added directly to the respective dishes, which were placed in the incubator. 24 h later, this step was repeated, and after another 24 h, cells were washed, trypsinized, and plated in 10 cm cell culture dishes with 2.0 μL/mL puromycin for selection.

### Radioactive tracer assay for lactate uptake

^14^C-lactate uptake was determined at RT. Cells were grown and differentiated in 6-well plates as described above, washed in isotonic Ringer solution (152 mM NaCl, 5 mM KCl, 1 mM Na_2_HPO_4_, 0.1 mM MgCl_2_, 1 mM CaCl_2_, 10 mM HEPES). Two separate wells were lysed in 1 M NaOH and used for determination of protein content in the wells using the Lowry method^47^ and bovine serum albumin dissolved in a final concentration of 1 M NaOH for generation of the protein standard curve. In remaining wells, influx was initiated at time 0, 1,2,3,4 and 4.5 min by adding 50 μL^14^C-lactate solution (final concentration 0.39 μM^14^C-lactate). Between time points, the plate was gently shaken to mix isotope and medium. Influx was terminated at time 5 min by removing the medium and washing in 2 mL PBS to removed remaining extracellular isotope. Following lysis in 96% ethanol, isotope was extracted in ddH_2_O, transferred to scintillation vials and activity determined by β-scintillation counting in Ultima Gold. Cellular^14^C-lactate activity in cpm/well was converted to nmol/g protein, using the extracellular specific activity (cpm/nmol) and the protein content (mg protein/well), and plotted as a function of time. The influx (nmol·g protein^−1^·min^−1^) was determined by linear regression.

### Seahorse analyzer measurements

Oxygen consumption rate (OCR) and extracellular acidification rate (ECAR) were assessed using a Seahorse XF96 Analyzer (Agilent). One-day post-confluent WT-1 cells were induced to differentiate, and were, on day 6, replated in gelatin-coated wells of an XF96 cell culture microplate. 100 μL of a 400.000 cells/ml cell suspension was added to each well. On day 8, cells were washed once with 150 μL of Seahorse medium (DMEM, Sigma-Aldrich D5030 containing 3 mg/ml phenol red, 32 mM NaCl_2_ and 5 mM glucose), another 180 µL medium was added to each well, and the microplate placed in the prep station. Inhibitors (AR-C, 4-CIN) and isoproterenol were diluted in Seahorse medium to a concentration 10-fold higher than the final concentration (final: 10 μM AR-C55858, 5 mM 4-CIN and 1 μM isoproterenol). Basal oxygen consumption rate (OCR) and extracellular acidification rate (ECAR) were measured first, followed by injection of isoproterenol, AR-C and 4-CIN to their respective wells at the times indicated. Data are shown as representative experiments or as mean with SD error bars, of 15–16 replicates per condition.

### Statistical analysis

Two-tailed, paired Students *t*-test, one- or two-way ANOVA with Bonferroni post-test were applied as relevant, with a p value of <0.05 taken to indicate a significant difference. Unless otherwise indicated, data are shown as representative individual experiments or as means with standard error of the mean (SEM) error bars, as indicated.

## Electronic supplementary material

Supplementary information
