# Supplementary material for: MCT1 and MCT4 Expression and Lactate Flux Activity Increase During White and Brown Adipogenesis and Impact Adipocyte Metabolism
Source: Sci Rep. 2017 Oct 12;7:13101. doi: 10.1038/s41598-017-13298-z (PMC5638914; doi:10.1038/s41598-017-13298-z)
Supplement: Supplementary file 1 — Supplementary information [file 41598_2017_13298_MOESM1_ESM.pdf]

Supplementary information for:

MCT1 and MCT4 Expression and Lactate Flux Activity Increase During White and Brown Adipogenesis and Impact Adipocyte Metabolism

Charlotte Petersen[1], Mette D. Nielsen[1], Elise S. Andersen[1,] Astrid L. Basse[1], Marie S. Isidor[1], Lasse K. Markussen[1], Birgitte M. Viuff[2], Ian H. Lambert[1], Jacob B. Hansen[1], and Stine F. Pedersen[1]

1) Section for Cell Biology and Physiology, Department of Biology, Faculty of Science, University of Copenhagen, Denmark, and 2) Section for Molecular Disease Biology, Department of Veterinary Disease Biology, Faculty of Health and Medical Sciences, University of Copenhagen, Denmark

Supplementary Figure 1

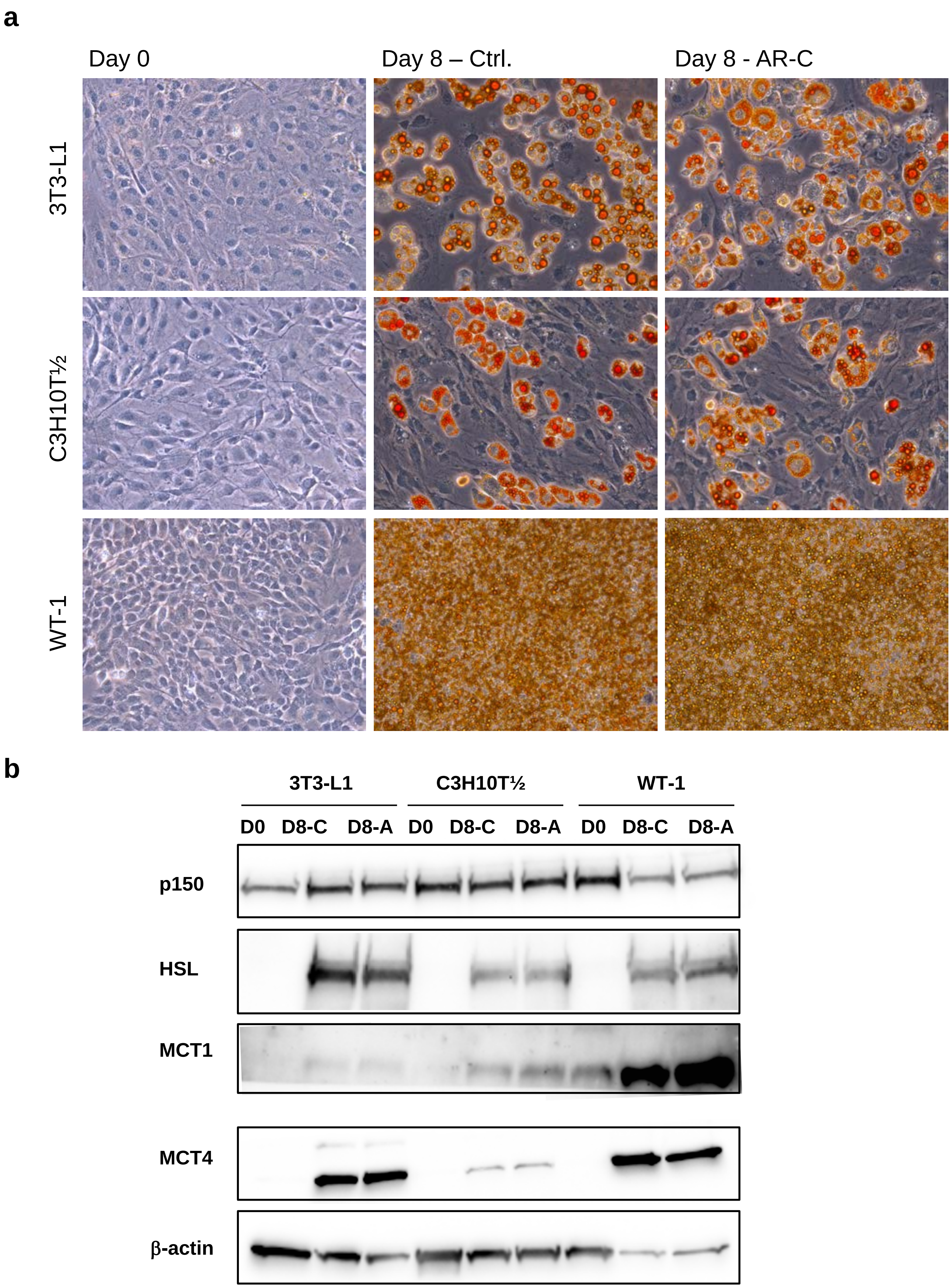

Supplementary Figure 2

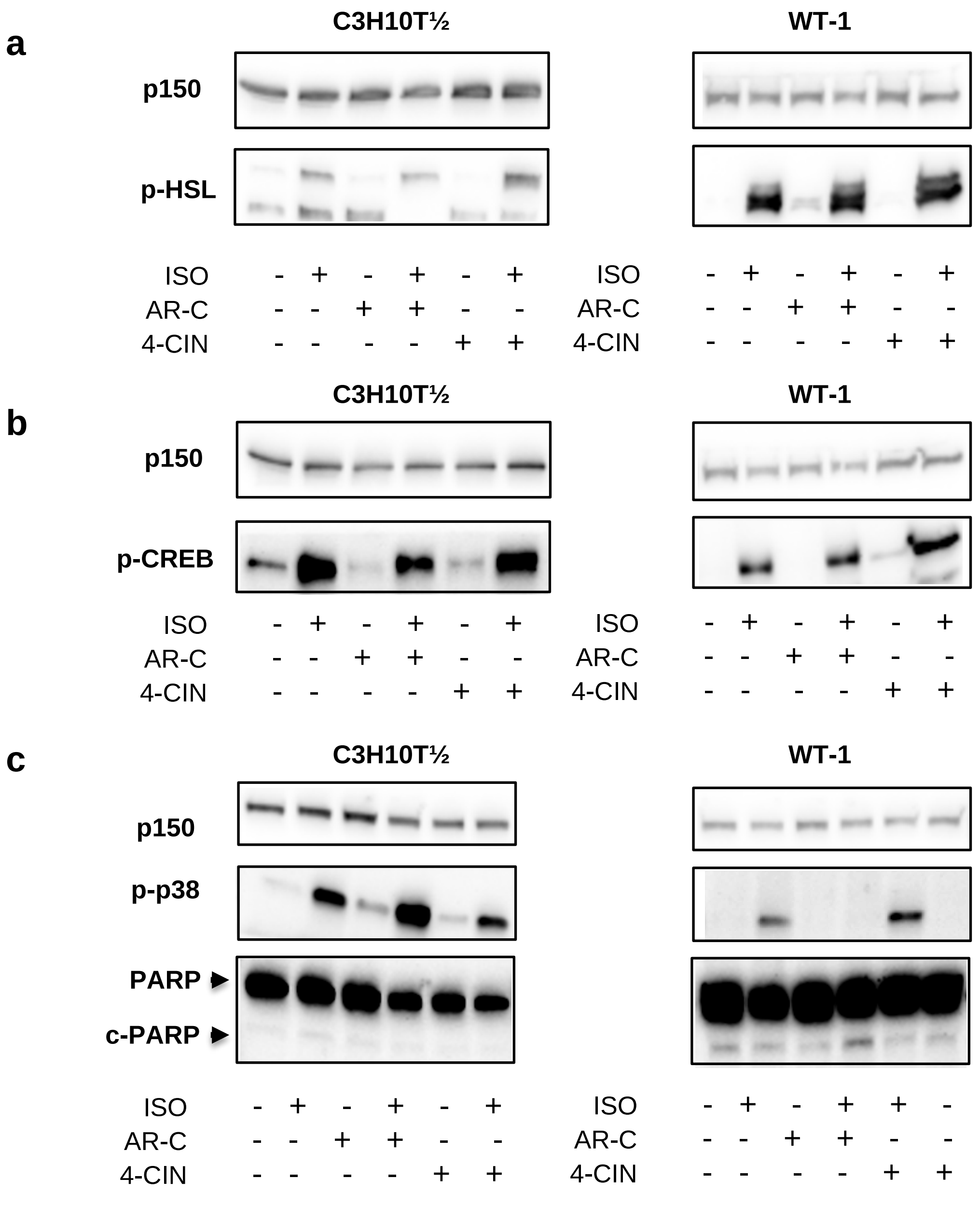

Legends to Supplementary Figures

Supplementary Figure 1. *Adipocyte differentiation is unaffected by inhibition of MCT1*

3T3-L1, C3H10T½, and WT-1 cells were differentiated as described in Materials and Methods, in the presence of AR-C (10 μM) or vehicle (DMSO) as indicated. a. To confirm differentiation, cells were stained with Oil Red O: dishes were washed twice in PBS and cells were fixed in 3.7 % formaldehyde for 1 h. After aspiration of the formaldehyde, the cells were stained with Oil Red O for 1 h. Oil red O was prepared by dissolving 0.5 g Oil Red O (Sigma-Aldrich) in 100 ml 2-propanol and diluting it with water (6:4), followed by filtration. Stained cells were washed carefully in PBS and covered with water until photographed using the 20X lens of a Leica DMI 6000 microscope. b. To further validate the differentiation, cells were differentiated as above, lysed, and subjected to immunoblotting for HSL, MCT1 and MCT4. p150 and β-actin are shown as loading controls. The data shown in both a and b are representative of three independent biological replicates per cell type. D0: day 0; D8-C: day 8, control (DMSO vehicle); D8-A: day 8, AR-C, 10 μM.

Supplementary Figure 2. *MCT inhibitors do not affect isoproterenol-induced cAMP signaling and cell integrity*

C3H10T½ and WT-1 cells were differentiated for 8 days and exposed to either AR-C (10 μM) or 4-CIN (5 mM) for 30 min, followed by exposure to isoproterenol (ISO, 1 μM) as a β-adrenergic agonist, for 15 min, as indicated. Cells were lysed and subjected to immunoblotting against (a) phospho-Ser660-HSL and (b) phospho-Ser133-CREB to evaluate cAMP-protein kinase A signaling, and (c) phospho-Thr180/Tyr182-p38 MAPK and PARP (full-length 116 kDa, and caspase-cleaved 89 kDa positions marked by arrows) to evaluate cell stress and apoptosis signaling. Note that the +/- ISO lanes are reversed in the 4-CIN condition in c. p150 is shown as loading control. The data shown in all three panels are representative of each three independent biological replicates per cell type.
